# Green Ultrasound-Assisted Extraction of Bioactive Compounds from Cumari-Do-Pará Peppers (*Capsicum chinense* Jacq.) Employing Vegetable Oils as Solvents

**DOI:** 10.3390/foods13172765

**Published:** 2024-08-30

**Authors:** Raiane Vieira Cardoso, Davi Vieira Teixeira da Silva, Samíria de Jesus Lopes Santos-Sodré, Patricia Ribeiro Pereira, Cyntia Silva Freitas, Diego Moterle, Luiz Alberto Kanis, Luiza Helena Meller da Silva, Antonio Manoel da Cruz Rodrigues, Vania Margaret Flosi Paschoalin

**Affiliations:** 1 Biochemistry Department, Chemistry Institute, Federal University of Rio de Janeiro (UFRJ), Avenida Athos da Silveira Ramos 149, Cidade Universitária, Rio de Janeiro 21941-909, RJ, Brazil; raiane.gnb@hotmail.com (R.V.C.); davivieiraufrj@gmail.com (D.V.T.d.S.); patriciarp@iq.ufrj.br (P.R.P.); freitas.cs@pos.iq.ufrj.br (C.S.F.); 2 Institute of Technology, Federal University of Para (UFPA), Augusto Corrêa 1, Guamá, Belém 66075-110, PA, Brazil; samiriasantos@gmail.com (S.d.J.L.S.-S.); lhmeller@ufpa.br (L.H.M.d.S.); amcr@ufpa.br (A.M.d.C.R.); 3 Health Science Institute, South University of Santa Catarina (UNISul), Avenida Jose Acacio Moreira 787, Tubarão 88704-900, SC, Brazil; diegomoterle@gmail.com (D.M.); luizalbertokanis@gmail.com (L.A.K.)

**Keywords:** green extraction, soybean and Brazilian nut oils, palm olein, vitamin C, capsaicin, carotenoids, phenolic compounds

## Abstract

Capsaicin, carotenoids, and phenolic compounds from cumari-do-Pará peppers (*Capsicum chinense* Jacq.) harvested from two different locations in Pará, Brazil, and at different ripening stages were extracted by employing green methodologies as an alternative to organic solvents. Edible vegetable oils from soybeans (*Glycine max*), Brazilian nuts (*Bertholettia excelsa* H.B.), and palm olein were used in combination with ultrasonic-assisted extraction (UAE). The proximate composition of the pepper extracts and vitamin C were determined through AOAC methods, total phenolics and carotenoids were assessed by UV/Vis spectrophotometry, and capsaicin by high-performance liquid chromatography. Antioxidant cumari-do-Pará extract activities were evaluated by the ABTS radical scavenging and β-carotene/linoleic acid assays. The vegetable oils were suitable for extracting and preserving bioactive pepper compounds, especially mature ones harvested from Igarapé-Açu. Bioactive compound content and antioxidant activity varied with harvesting location and ripening stage. Soybean oil was the most effective in extracting bioactive pepper compounds, particularly carotenoids, with 69% recovery. Soybean oil extracts enriched in capsaicin, carotenoids, and phenolics obtained from cumari-do-Pará can be used as spices in foodstuffs and/or as additives in pharmaceutical and nutraceutical formulations. Edible vegetable oils combined with UAE are promising for bioactive compound extraction, representing an environmentally friendly, safe, low-cost, versatile, and fast alternative.

## 1. Introduction

*Capsicum* spp. peppers, commonly known as cumari-do-Pará peppers, are very popular worldwide due to certain sensorial features, such as color, pungency, and aroma, also comprising a source of bioactive compounds that offer several health benefits. Adding pepper extracts to foods or consuming them on their own can boost antioxidant food power, as these extracts are a source of vitamins C and E, provitamin A, carotenoids, phenolic compounds, and, mainly, capsaicin [1,2,3,4,5]. Pepper consumption has been associated with significantly lower all-cause, cardiovascular, and cancer-related mortalities when consumed regularly, according to a meta-analysis evaluation, although a consumption regime has not yet been established [6]. In addition to interest due to flavor and sensorial aspects, capsaicin and other capsaicinoids found in peppers are also considered pharmacological agents, as they interact with the transient receptor potential cation channel subfamily V member 1 (TRPV1), which increases intracellular calcium levels and activates the sympathetic nervous system, releasing catecholamines [7,8,9,10]. These events increase and improve fat metabolism, thermogenesis, and blood glucose control, reducing the risk of obesity and metabolic syndrome, all of which underly the risk of cardiovascular events, strokes, and death [6,7,11,12]. Along with capsaicin, other compounds present in pepper extracts, such as vitamins C and E, provitamin A, carotenoids, and phenolic compounds, can also reduce cardiovascular and cancer-related complications, attenuating the cellular oxidative status by scavenging reactive oxygen and nitrogen reactive species and reducing inflammation through the nuclear factor erythroid 2-related factor 2 (Nrf2) and nuclear factor-kappaB (NFκB) signaling pathways [13,14,15,16,17]. Because of this, pepper products and extracts have been recognized both as food ingredients and potential pharmacological agents [18,19,20,21].

Traditionally, pepper processing aiming at obtaining bioactive compounds includes dehydration, milling, and conventional extraction employing organic solvents, such as methanol, ethanol, or petroleum-derived solvents [22,23,24]. However, organic solvents can be unsafe due to their residual presence in final products and the emission of potentially harmful volatile derivatives, which can affect the respiratory tract and may be neurotoxic or carcinogenic [25,26,27]. Furthermore, organic solvents are environmentally harmful pollutants that negatively affect the atmosphere and climate quality [28], and their removal from food and pharmaceutical additives is also time-consuming and costly [29,30,31,32].

Current regulations concerning non-environmentally friendly processes and restrictions imposed on food processing employing organic solvents have encouraged research on the application of green technologies to recover valuable compounds from edible plants through low-cost and safe processing methods [33,34,35,36]. In this sense, edible vegetable oils have been noted as promising green extraction solvents and have become popular for extracting bioactive compounds [37,38,39,40,41,42,43,44]. These oils are also renewable and non-toxic resources, as their methyl esters do not emit volatile organic compounds and exhibit comparable technical performances to organic solvents used in the extraction of bio-compounds from vegetable matrices [31,37,38,45]. The high viscosity of vegetable oils, however, is a major constraint concerning bioactive compound extraction due to low diffusivity and, consequently, low compound yields, even when applying high temperatures in the processing [40]. Ultrasonic-assisted extraction (UAE) has become a widely applied tool used to circumvent these and other limitations inherent to conventional extractions, i.e., extraction time and solvent consumption, while improving bioactive compound yields, particularly regarding highly hydrophobic compounds found in food matrices [31,33,34,40,46,47,48]. The UAE method is based on the cavitation generated by ultrasonic energy, which disrupts cell walls, allowing for highly efficient bioactive compound release and diffusion rates from vegetable matrices [31,33,34,40,46,47,48]. Finally, the quantity, quality, and yield of bioactive agents in vegetable matrices vary significantly depending not only on the cultivar, farming practices, and management, but mostly on the ripening stage [29,43,49,50].

Palm oil is the most produced, consumed, and traded oil in the world due to its versatility, production efficiency, and desirable properties, such as stability against oxidation, [51], while soybean oil is the vegetable oil most consumed in Brazil, mainly due to its costs and benefits [52]. Brazil nut oil is produced from native Amazon biome Brazil nuts [53,54]. They are important extractive species in the Amazon, and their commercialization is an important source of income for indigenous and riverine families living in the Brazilian Amazon rainforest. These edible vegetal oils were selected to be combined with UAE as sustainable alternatives to the organic solvents used to produce oily pepper extracts naturally enriched in bioactive compounds, representing an innovative, efficient, low-cost, safe, fast, and green methodology.

Edible vegetable oils used in the green extraction of bioactive compounds present significant advantages compared to organic solvents, as they are non-toxic and result in minimal environment and health impacts and the produced extracts do not require additional purification steps, as they are already suitable for human consumption or use. They may also be added to pharmaceuticals or health and hygiene products, comprising an advantageous alternative to the large-scale production of flavors or bioactive compounds. In this context, the suitability of vegetable oils combined with UAE to obtain bioactive compounds from cumari-do-Pará peppers (*Capsicum chinense* Jacq.) was investigated as an alternative to organic solvents. Peppers from different locations and maturation stages were used to expand the chances of good efficiency or high extraction yields.

## 2. Materials and Methods

### 2.1. Plant Material and Agroclimatic Study Area Characteristics

Peppers (*Capsicum chinense* Jacq.) were harvested from two different locations in the state of Pará, in Northern Brazil, within the Amazon rainforest biome. The first comprises Igarapé-Açu, at 01°07′44″ S, 47°37′12″ W, at an average altitude of 50.0 m a.s.l., with an annual average maximum temperature of 32.2 °C and relative humidity of 85%. Annual precipitation is high, reaching 2460 mm, strongly concentrated between January and June, while drier periods are noted between August and December [55,56]. Peppers were also harvested at Santo Antônio do Tauá at 01°09′07″ S, 48°07′46″ W, at an average altitude of 20.0 m a.s.l, with an annual average maximum temperature of 31.7 °C, relative humidity of 85%, and precipitation of 2600 mm, with the rainiest period occurring between December and May and a low hydric deficiency of 69 mm noted during drier periods between September and November. An annual water surplus of 1100 mm, referring to the maximum water soil retention, is noted in this area. The soil in the municipality of Santo Antônio do Tauá exhibits poor natural fertility, due to low essential nutrient and mineral contents [57]. Peppers were harvested in September 2016 at two different maturation stages, immature, comprising fully developed fruits just prior to the maturation onset, and mature, exhibiting a completely yellow skin. Over-mature and damaged peppers were discarded. For processing, peppers were sanitized in a 200 mg/L sodium hypochlorite solution for 15 min and washed with distilled sterilized water, and the seeds and pulps were ground, freeze-dried in a LS300 freeze-dryer (Terroni, SP, BRA) at −55 °C under a vacuum pressure of 55–100 µHg for 4 days, and immediately stored at −18 °C until use. The moisture content of freeze-dried peppers from different locations and ripening stages ranged from 8% to 10%.

The vegetable oils from soybeans or Brazilian nuts used to extract the bioactive pepper compounds were purchased from local stores in the city of Belém, Brazil (1°27′18″ S, 48°30′9″ W). Palm olein was donated by Agropalma, the largest sustainable palm oil producer in Latin America, established in the city of Belém, Brazil (https://www.agropalma.com.br/, accessed on 20 August 2024). The oils met quality standards concerning acidity < 0.6 mg KOH/g and the peroxide index < 10 meq/kg, among other specific requirements established by Resolution RDC No. 270, of 22 September 2005 [58].

### 2.2. Physicochemical Analyses

Centesimal composition analyses, namely moisture (method no. 932.12), protein (method no. 920.109), lipid (method no. 963.15), and ash (method no. 972.15), were carried out according to the Association of Official Analytical Chemists (AOAC) [59]. Total carbohydrates were estimated by subtracting the sum of moisture, protein, ash, and lipid content from 100%. pH values were determined using a pH-vision 246072 microcomputer (EXTECH Instruments, Waltham, MA, USA), while titratable acidity was expressed as a citric acid percent, and soluble solids were measured using an AR 1000S refractometer (Ionlab, PR, BRA), determining the refractive indices of each sample, reported in °Brix. Pepper vitamin C contents were determined in mg per 100 g, according to the AOAC method 43065 [60], where metaphosphoric acid, used as the solvent, was replaced by oxalic acid. All measurements were performed on fresh peppers in triplicate.

### 2.3. Fatty Acid Composition

The fatty acid composition of the employed vegetable oils was determined by their conversion into fatty acid methyl esters (FAMEs), according to Rodrigues et al. [61], through saponification with potassium hydroxide in methanol (0.1 mol·L^−1^) and esterification with hydrochloric acid in methanol (0.12 mol·L^−1^). The FAMEs were extracted with hexane (1:1 ratio) and analyzed using a CP-3380 gas chromatograph (Varian Inc., Palo Alto, CA, USA) equipped with a flame ionization detector (FID) and a CP-Sil 88 60 cm capillary column, with an internal diameter of 0.25 mm and film thickness of 0.25 µm (Varian Inc.). Helium was used as the carrier gas at a 0.9 mL/min flow rate, the FID detector and the injector (split ratio 1:100) were both set at 250 °C, and a 1.0 µL injection volume was used. The column temperature was set at 175 °C for 8 min, followed by a 2.0 °C/min increase up to 180 °C for 28 min, and a 2.0 °C/min increase up to 205 °C for 10 min. Individual fatty acids were identified and calculated by comparing their retention times and peak areas with the FAMEs standard reference Nucheck 74X (Nu-Chek Inc., Elysian, MN, USA) using the Varian Star 3.4.1 software (Varian Inc.). The results were expressed as fatty acids g·100 g^−1^ of sample.

### 2.4. Organic Solvent Extraction (OSE)

A traditional solvent extraction was applied for TPC, total carotenoids, and capsaicinoids, where the solvents were chosen considering the distinct solubilities and polar characteristics of each bioactive compound. Total phenolic compounds were extracted using an 70% aqueous acetone solution as described by Georgé et al. [62] and capsaicin was extracted with an 80% aqueous ethanol solution, according to Materska and Perucka [3]. Total carotenoids were extracted using two solvents, namely acetone for the initial extraction phase followed by petroleum ether, as described by Rodriguez-Amaya [63]. Concerning antioxidant capacity, 50% methanol and 70% acetone solutions were used as described by Rufino et al. [64].

### 2.5. Ultrasonic-Assisted Extraction (UAE)

Ultrasound-assisted extraction (UAE) was performed using a Maxiclean 800 ultrasonic bath device (Unique, SP, BRA) set to a constant power of 800 W and 20 kHz, according to Dias et al. [65], equipped with a digital control system for sonication time standardization. The freeze-dried pepper samples (1.0 g) were placed in 15 mL capped plastic tubes and individually mixed with 5 mL of oils obtained from soybeans, Brazil nuts, or palm olein, and sonicated for 60 min. Subsequently, the mixtures were centrifuged at 13,000× *g* for 20 min at 25 °C, and the supernatants, comprising the oily extract (OE), were separated for further analyses. Extraction parameters, mass:solvent ratios, and extraction times were established after preliminary tests.

### 2.6. Oily Extract (OE) Pretreatment

Prior to antioxidant activity, capsaicin, and TPC determinations, all OE underwent pretreatment by mixing 2 mL of each sample with 2 mL hexane and 10 mL methanol:water (50:50, *v*/*v*). The suspensions were then stirred by magnetic agitation at 1500× *g*, for 30 min at room temperature followed by methanol phase removal, filtering through 0.45 μm PVD filters (Merck Millipore, Burlington, MA, USA), and re-extraction. After two extractions, the methanol extracts were pooled and transferred to dark glass bottles, bubbled with nitrogen (N_2_), and stored in the dark at −20 °C until use.

### 2.7. Total Phenolic Content (TPC) Determinations

The TPC of the organic pepper solvent extracts (OSE) or OE were estimated using the Folin–Ciocalteu assay, according to Singleton et al. [66] and modified by Georgé et al. [62]. Briefly, 2.5 mL of the Folin–Ciolcateu reagent diluted in water (1/10) were added to pepper extracts obtained from OSE (Section 2.4) and to those acquired by OE after pretreatment (Section 2.6). After 2 min, 2 mL of a 7.5% sodium carbonate solution was added. The mixtures were then incubated for 15 min at 50 °C and finally cooled in an ice-water bath for 30 s. TPC was determined by measuring sample absorbances at 760 nm using a NI 2000 spectrophotometer (Novainstruments, Piracicaba, Brazil). TPC was expressed as gallic acid equivalents (GAE) mg·100 g^−1^ of sample.

### 2.8. Capsaicin Quantification

Capsaicin determinations were performed by high-performance liquid chromatography (HPLC) employing an LC-A10 Prominence (Shimadzu, Kyoto, Japan) apparatus equipped with a UV detector at 280 nm and a C18 column (150 mm × 4.6 mm × 5 µm). The mobile phase consisted of acetonitrile/water at a 60:40 ratio (*v*/*v*) and 1.0 mL/min flow rate at 40 °C, according to Perucka et al. [67], with modifications. Samples (40 µL) were injected into the HPLC system, and capsaicin was identified and quantified by comparison with capsaicin standards (Sigma-Aldrich Co., St. Louis, MO, USA). Data were expressed as mg of dry weight.

### 2.9. Total Carotenoid (TC) Determinations

Carotenoids from peppers were extracted by OSE, employing acetone and petroleum ether as the solvent mixture, while hexane at a 10:2 (*v*/*v*) ratio was used for the OE samples. Total carotenoids (TC) were determined by measuring sample absorbances at 450 nm on an NI 2000 spectrophotometer (Novainstruments, SP, BRA), according to Rodriguez-Amaya [63]. The results were expressed as µg of carotenoids per g of dry weight (µg lutein/g DW), using the molar extinction coefficient of lutein in petroleum ether (2589 M^−1^ cm^−1^).

### 2.10. Antioxidant Activity Determinations

#### 2.10.1. ABTS Radical Scavenging Assay

The ABTS^+^ assay was performed according to Rufino et al. [64]. The ABTS solution was prepared by mixing 5 mL of 7.0 µM ABTS and 88 μL of 145 µM potassium persulfate solution, which was left to react for 12–16 h at room temperature in the dark. Ethanol (99.5%) was added to the solution until reaching an absorbance value of 0.700 ± 0.05 at 734 nm, assessed using an NI 2000 UV/Vis spectrophotometer (Novainstruments, SP, BRA). Trolox (100–2000 µM TE) was used as the reference antioxidant compound. The ABTS solution was added to the samples, and the absorbances of both the samples and standard solutions were determined after 6 min at 734 nm at room temperature. Results were expressed as μmol Trolox equivalent (TE) g^−1^.

#### 2.10.2. β-Carotene/Linoleic Acid Determinations

The antioxidant activity of the obtained extracts was determined according to Matthäus [68] through the β-Carotene/linoleic acid assay, employing a model based on the generation of free radicals derived from methylene groups of linoleic acid, which in turn oxidize the double β-carotene bonds, promoting the rapid discoloration of the orange color in the absence of an antioxidant agent. A stock solution of a β-carotene-linoleic acid mixture was prepared by dissolving 3.34 mg β-carotene in 1 mL chloroform, with the addition of 40 mg linoleic acid and 400 mg Tween 20. The chloroform was completely evaporated, and 100 mL of water were added with vigorous shaking. Then, 5 mL of the mixture were dispensed in test tubes and a 200 µL aliquot of the extract (pretreated extract) was added and incubated at 50 °C for 60 min. Absorbances at 470 nm at time zero (A0) and after 60 min incubation (A60) were then determined using a NI 2000 UV-Vis spectrophotometer (Novainstruments, SP, BRA). Antioxidant activities were calculated according to the following equation:

(1) $$Antioxidant\;activity\left(\%\right)=\left[1-\frac{\left(A_{0}-A_{60}\right)}{\left(A_{0}^{c}-A_{60}^{c}\right)}\right]\times 100$$

where A_0_ and $A_{0}^{c}$ are the initial incubation time sample and control absorbances, respectively, while A_60_ and $A_{60}^{c}$ are the sample and control absorbances, respectively, determined at the end of the reaction.

### 2.11. Statistical Analyses

All determinations were performed in triplicate and expressed as means ± standard deviations (SD). The results were subjected to ANOVA and Pearson’s correlation tests, and potential differences between means were evaluated by Tukey’s multiple comparison test. Statistical significance was set at *p* < 0.05. All statistical analyses were performed using the Statistica^®^ 7.0 software (Statsoft Inc., Tulsa, OK, USA).

## 3. Results and Discussion

### 3.1. Proximate Composition and Bioactive Compound Analyses of in Natura Peppers

The physicochemical characteristics of immature and mature peppers from Taua and Igapare-Açú are presented in Table 1.

The influences of maturation stage and the origin of peppers on physicochemical parameters are displayed in Table 1. Pepper moisture contents varied from 87.76% to 91.46% for both maturation stages and locations, similar to those reported in peppers from other Capsicum genus members [5,69,70]. Moisture levels did not vary with maturation stage, with no differences observed between immature and mature peppers, although variations per geographic location were noted. In this sense, peppers from Tauá exhibited a higher moisture level, from 90.92 ± 0.17 to 91.46 ± 0.17 g·100 g^−1^, than those harvested at Igarapé-Açú, from 87.76 ± 0.45 to 87.90 ± 0.24 g·100 g^−1^. Tauá is located in a hydrographic region that includes several small rivers, such as Bituba, Caripé, Patauateua, São Francisco, and Tauá Rivers, and also receives the influence of the Sol and Furo da Laura bays that form a large water drainage network favoring agriculture and crop water capture [57]. Although moisture did not differ significantly between maturation stages, a trend of decreased moisture with increasing maturation stage was observed for the peppers from both locations. Moisture status affects maturation, as already described by Carvalho et al. [71], who reported moisture contents from 81.46% to 91.42% for immature peppers and from 78.19% to 89.39% for mature ones. Moisture losses during the ripening process may be related to respiratory cell functions or to the climatic characteristics inherent to the Amazon region, comprising high evapotranspiration demands combined with water losses to the atmosphere through plant surface evaporation and transpiration [72]. The peppers were harvested in September, during the local dry season and during one of the hottest months of the year, according to the National Institute for Space Research Weather Prevision Center and Climate Studies [73].

The total protein contents of peppers from Tauá varied between immature and mature specimens, with values of 1.22 ± 0.09 and 0.97 ± 0.22 g·100 g^−1^, respectively, while no protein content variation was observed between immature and mature peppers from Igarapé-Açú. Lower protein contents during ripening have been reported for other fruits, such as Barbados cherries (*Malpighia punicifolia* L.) [74]. Reduced protein contents during maturation can result from the breakdown of proteins into amino acids used to develop carbon skeletons for the synthesis of volatile compounds that contribute to aroma, which are enhanced in mature fruits [74]. On the other hand, Igarapé-Açú peppers exhibited protein content values of 1.49 ± 0.040 and 1.50 ± 0.026 g·100 g^−1^ in immature and mature fruits, respectively, higher than values observed in peppers from Tauá (Table 1). The previously reported low natural crop fertility, due to poor soil with low essential nutrient and mineral contents, may be related to the lower protein content found in Tauá fruits [57].

No significant difference in total lipid contents was observed between immature and mature peppers from Tauá (Table 1), in contrast to Igarapé-Açu peppers, where an increase in lipid contents from 0.94 ± 0.07 to 1.64 ± 0.06 g·100 g^−1^ in immature and mature fruits was observed. Increased phosphatidylcholine and phosphatidic acid contents during fruit ripening are due to increased respiratory rates, a characteristic of climacteric fruits [75].

Differences in ash contents were also noted between mature fruits from both locations (Tauá 0.71 ± 0.05 g·100 g^−1^ and Igarapé-Açu 0.94 ± 0.04 g·100 g^−1^), ranging from 0.65 ± 0.04 to 0.94 ± 0.04%, corroborating previous reports for Brazilian peppers, which indicated values ranging from 0.6 to 1.7% [76].

Regarding carbohydrates, differences were observed concerning ripening stages and geographic locations, as immature and mature Igarapé-Açu peppers exhibited higher carbohydrate contents compared with Tauá peppers, where the latter may suffer due to low soil nutrient content [77] (Table 1). However, during ripening, carbohydrate contents varied between locations, with a 12.3% increase in carbohydrate contents in mature peppers, ranging from 5.26 ± 0.21 to 5.91 ± 0.13 g·100 g^−1^, while peppers from Igarapé-açú presented an 8.5% carbohydrate content reduction from immature to mature fruits, from 8.91 ± 0.35 to 8.16 ± 0.69 g·100 g^−1^. Polysaccharides are mobilized during the aerobic metabolism during fruit maturation, increasing monosaccharide contents, such as glucose and fructose, which in turn improve fruit texture and flavor. This explains the higher sugar contents noted during Tauá pepper maturation [77]. On the other hand, the reduced sugar contents observed in Igarapé-Açú peppers may be due to fruit aging, as these peppers were probably harvested at the end of their life cycle, which may comprise a study limitation. In this sense, the anabolic metabolism was predominant in Igarapé-Açú peppers, resulting in higher consumption of simple sugars.

Soluble solid contents increased throughout pepper ripening at both sampled locations, with 8.5% and 11.5% increases observed in Tauá and Igarapé-Açu peppers, respectively. Increased soluble solids commonly occur during fruit ripening following the mobilization of starch and other polysaccharides from the cell wall, resulting in the monosaccharide accumulation alongside alkalization and consequent pH increases [78]. Similar soluble solid increases have been reported for Brazilian chilies (*Capsicum annuum* L. and “Zarco HS” Yellow) [79,80].

The titratable acidity of the investigated peppers varied for both maturation stage and geographical location, with 25% and 12.5% acidity level decreases observed in mature Tauá and Igarapé-Açu peppers, respectively, compared to immature peppers (Table 1). This is expected for *Capsicum* spp. during ripening, when the synthesis of organic acids occurs, with decreased organic acid contents noted at the end of the maturation period and beginning of senescence, as these compounds are consumed during the pepper respiratory metabolism [81,82].

Vitamin C contents decreased by approximately 40% in peppers harvested at both locations, associated with advanced ripening, from 128.16 ± 0.79 mg·100 g^−1^ in immature peppers to 77.15 ± 0.46 mg·100 g^−1^ in mature peppers from Tauá and from 101.91 ± 0.55 mg·100 g^−1^ in immature to 64.55 ± 0.36 mg·100 g^−1^ in mature peppers from Igarapé-Açu. Vitamin C decreases observed during fruit maturation may be due to ascorbic acid oxidase (ascorbinase) and peroxidase activities [82]. Similar results have been reported for sweet peppers and bell peppers (*C. annuum* L.), with vitamin C values ranging from 58.8 and 361.65 mg·100 g^−1^ in immature peppers and between 36.70 and 220 mg·100 g^−1^ in mature peppers [81,83,84]. Vitamin C confers resistance against biotic and abiotic stresses, and although the contents determined herein reduced with pepper ripening, the levels were still enough to meet the recommended dietary allowance of 60 to 90 mg/day for adults [85].

### 3.2. Total Phenolic Contents

Total phenolic compounds in both OSE and OE differed considering ripening stages and geographic locations (Table 2). Immature peppers presented lower phenolic compound concentrations compared to mature ones, and peppers from Igarapé-Açu contained the highest amounts of phenolic compounds (Table 2). The soil nutrient content from Tauá, characterized as of low fertility and poor, may have influenced the lower polyphenol content of this location [57].

Increased phenolic compound contents were observed, regardless of the extraction solvent, with advancing maturation in peppers harvested from both geographic locations (Table 2). Several studies have reported this trend, as many phenolic compounds are synthesized during the last fruit maturation stages [86,87]. The flavor and color of most mature fruits indicate increased phenolic compound contents at maturity, with polyphenols conferring several sensory and flavor characteristics to mature fruits [88].

Significant differences (*p* < 0.05) concerning extracted phenolic compounds were noted among the investigated extraction oils employed for pepper compound extraction. The OE/soybean was the most efficient, extracting the highest TPC (77.46 ± 1.69 and 113.58 ± 0.73 mg GAE 100 g^−1^) from mature peppers from both locations, respectively, compared to OE/Brazilian nut and OE/Palm olein. Considering maturation stages and geographic locations, mature peppers from Igarapé-Açu were of superior quality concerning TPC extraction. The extraction of phenolic compounds using vegetable oils as a solvent can be enhanced under intense extraction parameter optimization, as reported in studies of obtaining olive oils enriched with high TPC (414.3 ± 3.2 mg of oleuropein equivalent/kg of oil) [39,89]. Thus, vegetable oils have the potential to be used for TPC extraction, mainly for food purposes, compared to other lipophilic solvents.

This study indicates that vegetable oils can extract TPC, although in lower amounts (4–7% extraction efficiency) compared to OSE, considering both pepper maturation stage and geographic location (Table 2). It is important to note that significant polarity differences exist between organic solvents and vegetable oils. Phenolic compounds exhibit a higher affinity to polar solvents, such as aqueous acetone, used herein for OSE extraction, compared to non-polar and hydrophobic ones, such as vegetable oils, explaining the poor performance of vegetable oils regarding phenolic compound extraction from the investigated pepper matrices. On the other hand, edible vegetable oils, although resulting in lower phenolic compound extraction efficiency compared to organic solvents, are environmentally friendly and do not require an additional solvent evaporation step, due to their edible nature.

### 3.3. Capsaicin

Total capsaicin increased with advancing maturation in both geographic locations, higher in Igarapé-Açu peppers when employing both organic and oil solvents (Table 3). These findings corroborate previous data describing increasing capsaicin contents from the immature to mature stages in *Capsicum chinense* Jacq. “Habanero,” *Capsicum annuum* var. *acuminatum* L., and *Capsicum annuum* L. [43,50,90]. Higher capsaicin contents in Igarapé-Açu peppers (Table 3) may be associated with crop management characteristics, such as the applied irrigation regime, since deficient fruit hydration can result in increased capsaicinoid contents due to hydric stress, as reported in a study concerning habanero peppers subjected to water scarcity [91]. Furthermore, the time period between September and December at Igarapé-Açu is characterized by low rainfall rates, which may comprise a triggering factor for water stress responses, resulting in higher capsaicin synthesis and accumulation in peppers and other fruits from Northeastern Pará, Brazil [56].

OEs prepared from vegetable oils described herein differed from each other concerning capsaicin extraction yields, where OE/soybean seems to be the most efficient, able to extract higher amounts of capasaicin in the immature and mature stages (0.271 to 0.576 mg·g^−1^) compared to OE/Brazil nut (0.269 ± 0.04 to 0.500 ± 0.05 mg·g^−1^) and OE/palm olein (0.252 ± 0.03 to 0.496 ± 0.04 mg·g^−1^). Regarding ripening stage and locality, mature peppers exhibited higher capsaicin contents, especially those from Igarapé-Açu.

The capsaicin contents in OE concerning all pepper maturation stages and locations ranged from 0.252 ± 0.03 to 0.576 ± 0.03 mg·g^−1^, while contents in OSE with ethanol varied from 2.73 ± 0.02 to 6.13 ± 0.02 mg·g^−1^, probably due to capsaicin’s greater polarity and affinity to ethanol compared to vegetable oils (Table 3). Although the capsaicin recovery in OE samples was lower than that of OSE samples, the results achieved herein are superior to previous assessments using organic solvents. Capsaicin contents of 0.442 mg·g^−1^ in immature and 0.530 mg·g^−1^ in mature hot peppers (*C. annuum* L.) following ethanol extractions were reported by Materska and Perucka [3]. Other reports concerning *Capsicum chinese* extracted capsaicin ranging from 0.022 to 0.132 mg·g^−1^, 0.022 to 0.045 mg·g^−1^, 0.065 to 0.177 mg·g^−1^, and 0.020 mg·g^−1^ to 0.025 mg·g^−1^ when employing hexane, ethanol, acetone, and methanol as extraction solvents, respectively, combined with different traditional methods such as soxhlet extraction and maceration [92]. In addition, green extraction using vegetable oils obtained from mustard, sunflower, coconut, palm, and gingelly has achieved capsaicin extraction values between 0.01 and 0.02 mg·mL^−1^ through extraction by cooking *Capsicum chinense* peppers in the oils at 65 °C [93]. The results presented herein indicate that the edible vegetable oils used combined with UAE are promising for capsaicin extraction. However, further experimental conditions should be investigated aiming for the optimization of the green extraction method to achieve higher yields.

### 3.4. Total Carotenoids

Total carotenoid contents increased with increasing pepper maturation stage and differed by location (Table 4). Extracts from peppers, especially mature ones, from Igarapé-Açu contained the highest concentration of extracted carotenoids compared to those from Tauá, maybe due to the aforementioned poor soil quality [57]. Increases in total carotenoid compound contents in ripening peppers have been reported in other assessments. Reduced chlorophyll content occurs during ripening, with increased carotenoid synthesis, responsible for fruit coloring [49,50,87,94]. Herein, the soybean oily pepper extracts contained the highest total carotenoid yields, followed by OE/Brazilian nut and OE/palm olein (Table 4). Although oily extract recoveries were again inferior to those following organic solvent extraction, a 69% recovery could be achieved when compared to the OSE when soybean oil and mature peppers from Igarapé-Açu were used to prepare the oily extract (Table 4).

The efficiency of the employed oils in extracting bioactive pepper compounds compared to organic solvents reveals different extraction performances between oils, with soybean oil standing out as the most efficient, regardless of the sample. For Tauá peppers, the extraction efficiency ranged from 86.12% to 78.93% when using soybean oil, from 63.25% to 54.27% using Brazilian nuts, and from 57.62% to 49.35% employing palm olein, considering immature and mature peppers, respectively. Concerning Igarapé-Açu peppers, extraction efficiencies were 76.52–69.20% when using soybean oil, 60.71–55.96% using Brazilian nuts, and 58.04–50.77% when employing palm olein, for immature and mature peppers, respectively. This indicates that soybean oil not only provides greater extraction efficiency but also maintains superior performance for both pepper maturation stages. This can be attributed to its physicochemical properties, such as viscosity and fatty acid profile. Furthermore, the observed extraction efficiency variations between oils and samples (immature versus mature) may reflect pepper chemical composition and structure differences, which influence target compound solubility. The decreased efficiency observed when using Brazilian nut and palm olein suggests that these solvents have a lower capacity to penetrate or dissolve the compounds present in the pepper samples [38,95,96].

The replacement of organic solvents with vegetable oils can find potential applications and advantages, as they are safe (non-toxic) and natural (vegetable origin), can act as a barrier between the carotenoids extracted in the vegetable oil and the atmosphere, avoiding oxidation during the manipulation and processing, and can increase the yields of extraction of lipid molecules due to the lipophilic nature of vegetable oils [38,97].

The higher extraction efficiency of vegetable oils is probably due to the lipophilic character of carotenoid compounds, which, combined with UAE, results in greater carotenoid diffusion from the pepper matrix to the lipid solvent. Besides the well-known potential of carotenoids in the prevention of vitamin A deficiency, they have also been reported as contributing to the prevention of numerous chronic oxidative and age-related illnesses, including cancer, heart disease, and macular degeneration, due to their antioxidative capacity [98]. A meta-analysis investigation including randomized controlled trials and observational studies comprising 28,944 overweight and obese individuals indicated a significant association between carotenoid intake (1.2–60 mg d^−1^) and body weight reduction, body mass index decreases, and waist circumference losses [99]. It is widely known that obesity and overweightness comprise a risk factor for cardiovascular disease, one of the leading causes of death worldwide [100].

### 3.5. Fatty Acid Vegetable Oil Profiles

The TPC, capsaicin, and total carotenoid compound analyses of cumari-do-Pará peppers (Table 2, Table 3 and Table 4) indicated that the most effective vegetable oil for bioactive compound extraction was soybean oil. Herein, the composition of the primary fatty acids found in soybean oil, Brazilian nut oil, and palm olein present significant amounts of linoleic acid (ω-6), oleic acid (ω-9), and palmitic acid, representing around 90% of total essential fatty acids (Table 5). Minor fatty acids are also reported, including linolenic acid, stearic acid, arachidic acid, stearic acid, lauric acid, and myristic acid, reaching around 10% of total fatty acids [54,101,102]. The fatty acid profile of soybean oil exhibits a higher percentage of unsaturated fatty acids, such as linoleic and oleic acid (Table 5), which could contribute to the greater efficiency in extracting hydrophobic bioactive compounds. Polyunsaturated fatty acids such as linoleic acid tend to present lower viscosity than monounsaturated and saturated fatty acids. Kim et al. [103] observed a decrease in the viscosity of vegetable oils, clearly influenced by increases in the amount of polyunsaturated fatty acids (18:2) and decreases in monounsaturated fatty acids (18:1). Double bonds do not allow fatty acid molecules to stack together, interfering with their crystalline packing. Therefore, unsaturated fatty acids do not have a rigid and fixed structure, being less compacted and more fluid, consequently interfering with viscosity. Vegetable oils with lower viscosity affect diffusivity by decreasing the interactions between the solution and solvent molecules [40,45,104].

Thus, employing high viscosity oils may result in poor extraction of and consequently lower bioactive compound yields [38]. This was verified herein, as different extraction efficiencies were noted and can be input to the differences in viscosities of the investigated oils, where palm olein presents the highest viscosity (33.79 mPa-s), followed by Brazilian nut oil (31.86 mPa-s) and, finally, soybean oil (29.5 mPa-s) [105,106,107]. In this regard, extraction performed with soybean oils corroborates the aforementioned discussion, allowing for OE enrichment with TPC, capsaicin, and, primarily, carotenoids. It is important to note that in addition to the bioactive pepper compounds, soybean oil will still provide oleic and linoleic acids, which are also strongly associated with cardiovascular disease prevention and other health benefits, as mentioned previously [108,109].

Good storage stability of soybean oil has already been reported, which demonstrated no changes in fatty acids during 60 days of storage under different conditions, such as oxygen presence, temperatures up to 32 °C, and cold fluorescent light [110]. The degree of saturation remained unchanged in soybean oils even in the presence of oxidative conditions. Herein, the oily extracts are enriched by blending antioxidant compounds, recognized as a dynamic strategy to obtain oils with excellent storage stability and stable fatty acid composition [111,112,113,114]. Vegetable oils as a solvent provide a protective barrier against the attraction of oxygen molecules, delaying the oxidation and degradation time of extracts of bioactive compounds, such as carotenoids [38,40,42].

### 3.6. Antioxidant Activity Determined by the ABTS and β-Carotene/Linoleic Acid System Assays

Pepper extract antioxidant activities evaluated by the ABTS and β-carotene/linoleic acid system assays followed the same trend as observed for the extracted bioactive compound contents, revealing differences according to pepper ripening stage, with a tendency towards higher antioxidant activity in mature peppers, corroborating previous reports (Table 6) [1,49]. Furthermore, several biochemical changes occur in response to biotic and abiotic factors throughout the fruit maturation process, culminating in the synthesis and increased content of new phytochemicals and consequently greater antioxidant capacity.

As demonstrated by the Pearson correlation coefficient, bioactive compounds found in the OSE were positively correlated with antioxidant activity determined by the ABTS method (r = 1.00) and the β-carotene/linoleic acid system (r = 0.97) assays (Table S1). The same correlation was also established in other studies [3,50,71,87,115]. Concerning OE, several compounds, i.e., capsaicin, phenolic compounds, and carotenoids, increased during the pepper ripening process, positively influencing antioxidant activity inferred by the two methods (r > 0.78) (Table S1). This proves that antioxidant activity is not dependent on a single compound but on several, as well as their interactions [81,90]. Moreover, besides being capable of extracting bioactive pepper compounds, the oily solvents preserved their functional characteristics, corroborated by their antioxidant activity, reaching 40% antioxidant activity recovery compared to the solvent extracts (Table 6).

Vegetable oils are important from a technological point of view, as their oxygen barrier properties slow down the oxidizing and degrading processes of the extracted compounds, eliminating the need for the evaporation or solvent separation steps usually included in OSE preparation [89].

Many studies show that oily solvent extractions are able to achieve yields similar to those obtained with organic solvents [89]. Further studies are in progress to optimize these extractions, especially concerning phenolic compounds and capsaicin, from peppers using soybean oil. Different strategies should be investigated, including pepper:oil ratios (*w*/*v*), extraction times, ultrasound parameters, and temperature. The use of different techniques is not discarded, such as high-pressure processing, microwave, and supercritical CO_2_ frequently associated with green extraction, [89]. Under optimized conditions, studies can progress towards detailed experiments such as bioassays to test the biological activities associated with the bioactive compounds present in OE, as well as the characterization of the phenolic compound profiles present in these enriched edible oils.

### 3.7. A Brief Overview on the Costs and Benefits of Organic Solvent Extraction Compared to Vegetable Oils in Obtaining Bioactive Compounds

The use of organic solvents requires a subsequent evaporation step that can result in the loss of bioactive compounds, as many of them are thermally labile. The high temperatures required for solvent evaporation can also degrade bioactive compounds. As stated previously, the Soxhlet extraction method, for example, involves prolonged exposure to heat and degradation of heat-labile compounds [116,117]. Although few studies explore the loss of bioactive compounds pre- and post-solvent evaporation, the impact of thermal processing, necessary for solvent extraction and removal, has been reiterated in several reviews, especially for phenolic compounds [118]. Phenolic compounds were found to be strongly affected by the 70% methanol solution when the evaporation was preceded by rotary evaporation at 40 °C for 6 h [119].

During solvent evaporation by rotary evaporators, volatile compounds can evaporate along with the solvent, resulting in a dry extract and cause antioxidant activity losses, even though an apparent higher OSE efficiency is noted compared to vegetable oils [116,117,119]. In this sense, the bioactive compounds content found in the OSE may not reflect reality, since this is not subjected to subsequent solvent evaporation. In contrast, when vegetable oils are employed as solvents, the OEs are already ready to use.

## 4. Conclusions

The use of vegetable oils in the extraction of bioactive pepper compounds is simple and feasible. The combination of UAE and edible vegetable oils does not exhibit the same extraction efficiency as the organic solvents investigated herein, probably due to the low lipophilicity of certain bioactive compounds such as polyphenols with a maximum efficiency of 7%, while carotenoids, which display high hydrophobicity, were more prone to diffusion from the pepper matrix to the vegetable oils, reaching maximum extraction of 86%. On the other hand, the bioactive contents of the vegetable oil extracts were close to or higher than those found in organic solvent extracts reported in previous assessments. The use of vegetable oils associated with UAE allows for ready-to-use preparations of edible bioactive compounds and unsaturated fatty acid-rich extracts obtained from peppers. Furthermore, no additional solvent evaporation step is required, unlike for organic solvents, avoiding bioactive and/or flavor losses and potential toxic subproducts that may negatively affect human health or the environment. Edible oils, such as soybean oil, which exhibited the greatest extraction efficiency, are cheap, widely consumed, and commercialized in Brazil, allowing the acceleration of the industrial production of these novel pepper extracts, both environmentally friendly and beneficial for human health.

The physicochemical characteristics, bioactive compound contents, and antioxidant activities of the oily extracts prepared from mature and immature *Capsicum chinense* Jacq. were assessed herein. The beneficial power of the oily pepper extracts was influenced by unfavorable crop conditions, such as low soil hydration and low soil fertility. Both may affect pepper moisture levels and bioactive compound contents in response to abiotic stresses, although fruit ripening is associated with increased bioactive compound contents and antioxidant activities. However, fruits must be harvested under satisfactory farming conditions, such as adequate hydration levels and favorable nutrition soil and climate conditions. A limitation of this study is the fact that no systematic or instrumental fruit ripening degree control (unripe, medium ripe, ripe, and overripe) was applied, and fruits were categorized only as immature or mature peppers and not harvested after the same number of days.

## Figures and Tables

**Table 1:** Proximate composition of immature and mature cumari-do-Pará peppers from two locations, Tauá and Igaparé-Açu in the state of Pará, Brazil.

Analyses	TAUÁ		IGARAPÉ-AÇU	
	Immature	Mature	Immature	Mature
Moisture (g·100 g^−1^)	91.46 ± 0.17 ^a^	90.92 ± 0.17 ^a^	87.90 ± 0.24 ^c^	87.76 ± 0.45 ^c^
Total proteins (g·100 g^−1^)	1.22 ± 0.09 ^b^	0.97 ± 0.22 ^c^	1.49 ± 0.04 ^a^	1.50 ± 0.026 ^a^
Total lipids (g·100 g^−1^)	1.41 ± 0.09 ^a^	1.49 ± 0.05 ^a^	0.94 ± 0.07 ^b^	1.64 ± 0.06 ^c^
Ashes (g·100 g^−1^)	0.65 ± 0.04 ^b^	0.71 ± 0.05 ^b^	0.76 ± 0.05 ^ab^	0.94 ± 0.04 ^a^
Carbohydrate (g·100 g^−1^)	5.26 ± 0.21 ^d^	5.91 ± 0.13 ^c^	8.91 ± 0.35 ^a^	8.16 ± 0.69 ^b^
Soluble solids (°Brix)	8.57 ± 0.26 ^d^	9.30 ± 0.06 ^b^	9.2 ± 0.00 ^c^	10.26 ± 0.06 ^a^
Titratable acidity (% citric acid)	0.45 ± 0.01 ^a^	0.34 ± 0.01 ^c^	0.40 ± 0.01 ^b^	0.35 ± 0.00 ^c^
pH	5.07 ± 0.00 ^c^	5.25 ± 0.02 ^b^	5.33 ± 0.02 ^a^	5.36 ± 0.00 ^a^
**Bioactive compounds**				
Vitamin C (mg·100 g^−1^)	128.16 ± 0.79 ^a^	77.15 ± 0.46 ^c^	101.91 ± 0.55 ^b^	64.55 ± 0.36 ^d^
Total phenolic content (mg GAE·100 g^−1^)	697.64 ± 5.36 ^d^	757.49 ± 6.39 ^b^	724.69 ± 6.42 ^c^	825.21 ± 3.32 ^a^
Capsaicin (mg·g^−1^)	1.42 ± 0.01 ^d^	1.77 ± 0.00 ^c^	3.12 ± 0.01 ^b^	3.26 ± 0.01 ^a^
Total carotenoids (μg·g^−1^)	11.43 ± 0.07 ^d^	22.97 ± 0.08 ^b^	17.17 ± 0.01 ^c^	29.41 ± 0.06 ^a^
**Antioxidant analyses**				
β-carotene/linoleic acid (%AA)	24.48 ± 0.46 ^c^	27.26 ± 0.48 ^b^	27.06 ± 0.27 ^b^	31.77 ± 0.49 ^a^
ABTS (µM trolox·g^−1^)	46.79 ± 1.67 ^c^	50.23 ± 0.47 ^b^	50.17 ± 1.98 ^b^	65.83 ± 2.20 ^a^

The results are presented on a wet weight basis following three determinations (mean ± standard deviation). Same letters on the same line indicate no difference between maturation stages and/or locations according to the Tukey test (*p* ≤ 0.05).

**Table 2:** Total phenolic contents of OSE and OE from peppers obtained from Tauá and Igarapé-Açu, Pará, Brazil.

Total Phenolic Contents (mg GAE 100 g^−1^)	TAUÁ		IGARAPÉ-AÇU	
	Immature	Mature	Immature	Mature
OSE/acetone 70%	1332.25 ± 8.02 ^Ca^	1435.23 ± 17.73 ^Ba^	1367.27 ± 12.88 ^Ca^	1542.21 ± 8.02 ^Aa^
OE/Soybean	53.02 ± 0.73 ^Db^	77.46 ± 1.69 ^Cb^	87.10 ± 0.71 ^Bb^	113.58 ± 0.73 ^Ab^
OE/Brazilian nut	52.05 ± 0.62 ^Db^	70.18 ± 1.04 ^Cc^	85.99 ± 1.54 ^Bb^	106.98 ± 0.62 ^Ac^
OE/Palm olein	51.08 ± 0.43 ^Db^	67.73 ± 1.81 ^Cc^	80.01 ± 0.89 ^Bc^	100.56 ± 0.43 ^Ad^

Results are presented on a dry weight basis following triplicate extractions and determinations (mean ± standard deviation). Means followed by the same capital letters in lines indicate no significant difference between maturation stage and geographic location, while the same lowercase letters in columns indicate no significant difference between extraction solvents according to the Tukey test (*p* ≤ 0.05). OSE—organic solvent extract, OE—oily extract.

**Table 3:** Total capsaicin in OSE and OE from peppers harvested in Tauá and Igarapé-Açu, Pará, Brazil.

Capsaicin (mg·g^−1^)	TAUÁ		IGARAPÉ-AÇU	
	Immature	Mature	Immature	Mature
OSE/ethanol	2.73 ± 0.02 ^Da^	3.38 ± 0.01 ^Ca^	5.86 ± 0.03 ^Ba^	6.13 ± 0.02 ^Aa^
OE/Soybean	0.271 ± 0.03 ^Db^	0.394 ± 0.06 ^Cb^	0.492 ± 0.05 ^Bb^	0.576 ± 0.03 ^Ab^
OE/Brazil nut	0.269 ± 0.04 ^Dc^	0.354 ± 0.04 ^Cc^	0.482 ± 0.01 ^Bc^	0.500 ± 0.05 ^Ac^
OE/Palm olein	0.252 ± 0.03 ^Dd^	0.273 ± 0.01 ^Cd^	0.479 ± 0.08 ^Bd^	0.496 ± 0.04 ^Ad^

Capsaicin contents from three distinct extractions are reported on a dry weight basis following triplicate determinations (mean ± standard deviation). Means followed by the same capital letters in lines indicate no significant difference between maturation stage and location, while the same lowercase letters in columns indicate no significant difference between samples concerning each investigated solvent according to the Tukey test (*p* ≤ 0.05). OSE—organic solvent extract, OE—oily extract.

**Table 4:** Total carotenoid contents in OSE and OE from peppers harvested in Tauá and Igarapé-Açu, Pará, Brazil.

Total Carotenoids (μg·g^−1^)	TAUÁ		IGARAPÉ-AÇU	
	Immature	Mature	Immature	Mature
OSE/acetone, petroleum ether	21.90 ± 0.14 ^Da^	43.87 ± 0.16 ^Ba^	32.25 ± 0.30 ^Ca^	55.23 ± 0.12 ^Aa^
OE/Soybean	18.86 ± 0.17 ^Db^	34.63 ± 0.11 ^Bb^	24.68 ± 0.19 ^Cb^	38.22 ± 0.16 ^Ab^
OE/Brazilian nut	13.77 ± 0.12 ^Dc^	23.81 ± 0.17 ^Bc^	19.58 ± 0.22 ^Cc^	30.91 ± 0.06 ^Ac^
OE/Palm olein	12.62 ± 0.17 ^Dd^	21.65 ± 0.23 ^Bd^	18.72 ± 0.36 ^Cd^	28.04 ± 0.28 ^Ad^

The results are presented on a dry weight basis following triplicate extractions and determinations (mean ± standard deviation). Means followed by the same capital letters in lines indicate no significant difference between maturation stages and location, while the same lowercase letters in columns indicate no significant difference between samples concerning each extraction solvent according to the Tukey test (*p* ≤ 0.05). OSE—organic solvent extract, OE—oily extract.

**Table 5:** Major fatty acids found in palm olein, Brazilian nut oil, and soybean oil.

Vegetable Oil	Fatty Acid (g·100 g^−1^)		
	Palmitic Acid (C16:0)	Oleic Acid (C18:1, ω-9)	Linoleic Acid (C18:2, ω-6)
Soybean	12.40 ± 0.05 ^c^	23.30 ± 0.05 ^c^	58.21 ± 0.07 ^a^
Brazilian nut	15.25 ± 0.03 ^b^	38.48 ± 0.02 ^b^	35.87 ± 0.02 ^b^
Palm olein	37.81 ± 0.10 ^a^	44.62 ± 0.09 ^a^	10.70 ± 0.12 ^c^

Different letters in the same column indicate significant differences according to the Tukey test (*p* ≤ 0.05).

**Table 6:** Antioxidant activity of OSE and OE evaluated by the ABTS and β-carotene/linoleic acid assays from peppers harvested at Tauá and Igarapé-Açu, Pará, Brazil.

ABTS (µM Trolox g^−1^)	TAUÁ		IGARAPÉ-AÇU	
	Immature	Mature	Immature	Mature
OSE/methanol, acetone	89.58 ± 1.73 ^Ca^	95.89 ± 0.77 ^Ba^	94.27 ± 1.30 ^Ba^	123.61 ± 2.61 ^Aa^
OE/Soybean	16.08 ± 0.26 ^Db^	24.05 ± 0.50 ^Cb^	26.44 ± 0.18 ^Bb^	42.45 ± 0.68 ^Ab^
OE/Brazil nut	13.57 ± 0.14 ^Cbc^	22.84 ± 0.21 ^Bb^	24.16 ± 0.50 ^Bbc^	40.50 ± 0.35 ^Abc^
OE/Palm olein	10.56 ± 0.11 ^Cc^	21.17 ± 0.44 ^Bb^	22.92 ± 0.30 ^Bc^	39.12 ± 0.45 ^Ac^
**β-Carotene/Linoleic Acid** **%AA (60 min)**	**TAUÁ**		**IGARAPÉ-AÇU**	
	**Immature**	**Mature**	**Immature**	**Mature**
OSE/methanol, acetone	46.88 ± 0.79 ^Ca^	52.06 ± 0.80 ^Ba^	50.86 ± 0.51 ^Ba^	59.66 ± 0.92 ^Aa^
OE/Soybean	9.24 ± 0.19 ^Db^	16.20 ± 0.40 ^Bb^	14.97 ± 0.33 ^Cb^	24.09 ± 0.51 ^Ab^
OE/Brazil nut	7.90 ± 0.32 ^Dbc^	14.82 ± 0.19 ^Bbc^	12.25 ± 0.54 ^Cc^	22.18 ± 0.83 ^Ab^
OE/Palm olein	6.70 ± 0.38 ^Cc^	13.22 ± 0.19 ^Bc^	11.34 ± 0.57 ^Bc^	19.08 ± 0.24 ^Ac^

The results are presented on a dry weight basis following triplicate extractions and determinations (mean ± standard deviation). Means followed by the same capital letters in the same line indicate no significant difference between the maturation stage and harvesting location, while the same lowercase letters in columns indicate no significant difference between samples in each extraction solvent according to the Tukey test (*p* ≤ 0.05). %AA—percentage of antioxidant activity, OSE—organic solvent extract, OE—oily extract.

## Data Availability

The original contributions presented in the study are included in the article/Supplementary Material, further inquiries can be directed to the corresponding author.

## Supplementary Materials

The following supporting information can be downloaded at https://www.mdpi.com/article/10.3390/foods13172765/s1: Table S1: Pearson’s correlation coefficient (r) between bioactive compounds and the antioxidant capacity of pepper extracts obtained with organic solvents and vegetable oils.
