# Supplementary material for: Green Ultrasound-Assisted Extraction of Bioactive Compounds from Cumari-Do-Pará Peppers (Capsicum chinense Jacq.) Employing Vegetable Oils as Solvents
Source: Foods. 2024 Aug 30;13(17):2765. doi: 10.3390/foods13172765 (PMC11394977; doi:10.3390/foods13172765)
Supplement: Supplementary file 1 [file foods-13-02765-s001.zip › foods-3138634-supplementary.pdf]

Table S1. Pearson's correlation coefficient (r) between bioactive compounds and the antioxidant capacity of pepper extracts obtained with organic solvents and vegetable oils.

Pepper extracts	Bioactive compounds	ABTS (µM trolox g^{-1})	β-carotene/linoleic acid %AA (60 min)
		r	
Organic solvent	Vitamin C (mg.100 g^{-1})	-0.79	-0.90
	Total phenolic (mg GAE.100 g^{-1})	0.97*	1.00*
	Total carotenoids (µg.g^{-1})	0.61	0.74
	Capsaicin (mg.g^{-1})	0.81	0.70
Vegetable oils	Total phenolic (mg GAE.100g)	0.97*	0.93*
	Total carotenoids (µg.g^{-1})	0.78*	0.85*
	Capsaicin (mg.g^{-1})	0.86*	0.79*

Tukey test ($p \leq 0.05$) r= Pearson's correlation coefficient
*Significant static correlation between the bioactive compound and antioxidant activity.
Negative values demonstrate a negative correlation, and positive values demonstrate a positive correlation. The closer to r = 1, the greater the association between bioactive compounds and antioxidant activity (ABTS and β-carotene/linoleic acid).
